# Maternal–Infant Supplementation with Small-Quantity Lipid-Based Nutrient Supplements Does Not Affect Child Blood Pressure at 4–6 Y in Ghana: Follow-up of a Randomized Trial

**DOI:** 10.1093/jn/nxy285

**Published:** 2019-02-11

**Authors:** Sika M Kumordzie, Seth Adu-Afarwuah, Rebecca R Young, Brietta M Oaks, Solace M Tamakloe, Maku E Ocansey, Harriet Okronipa, Elizabeth L Prado, Kathryn G Dewey

**Affiliations:** 1 Program in International and Community Nutrition, Department of Nutrition, University of California Davis, Davis, CA; 2 Department of Nutrition and Food Science, University of Ghana, Legon, Ghana; 3 Department of Nutrition and Food Sciences, University of Rhode Island, Kingston, RI

**Keywords:** supplementation, blood pressure, lipid-based nutrient supplements, Ghanaian children; prenatal; infant nutrition

## Abstract

**Background:**

In the International Lipid-Based Nutrient Supplements (iLiNS)-DYAD-Ghana trial, prenatal small-quantity lipid-based nutrient supplements (LNSs) had a positive effect on birth weight. Birth weight may be inversely related to blood pressure (BP) later in life.

**Objectives:**

We examined the effect of the intervention on BP at 4–6 y of age, and maternal and child factors related to BP.

**Methods:**

The iLiNS-DYAD-Ghana study was a partially double-blind, randomized controlled trial which assigned women (*n* = 1320) ≤20 weeks of gestation to daily supplementation with: *1*) iron and folic acid during pregnancy and 200 mg Ca for 6 mo postpartum , *2*) multiple micronutrients during pregnancy and postpartum, or *3*) LNSs during pregnancy and postpartum plus LNSs for infants from 6 to 18 mo of age. At 4–6 y of age (*n* = 858, 70% of live births), we compared BP, a secondary outcome, between non-LNS and LNS groups and examined whether BP was related to several factors including maternal BP, child weight-for-age *z* score (WAZ), and physical activity.

**Results:**

Non-LNS and LNS groups did not differ in systolic (99.2 ± 0.4 compared with 98.5 ± 0.6 mm Hg; *P* = 0.317) or diastolic (60.1 ± 0.3 compared with 60.0 ± 0.4 mm Hg; *P* = 0.805) BP, or prevalence of high BP (systolic or diastolic BP ≥90th percentile of the US National Heart, Lung, and Blood Institute reference: 31% compared with 28%; *P* = 0.251). BP at 4–6 y of age was positively related to birth weight; this relation was largely mediated through concurrent WAZ in a path model. Concurrent WAZ and maternal BP were the factors most strongly related to child BP.

**Conclusions:**

Despite greater birth weight in the LNS group, there was no intervention group difference in BP at 4–6 y. In this preschool population at high risk of adult hypertension based on BP at 4–6 y, high maternal BP and child WAZ were key factors related to BP. This trial was registered at clinicaltrials.gov as NCT00970866.

## Introduction

The maternal intrauterine environment plays a key role in fetal development and conditions the offspring for risk of certain metabolic diseases later in life (1). Because fetal growth is strongly influenced by nutrient and oxygen supply in utero, prenatal nutrition is considered a key programming stimulus (2). There is considerable evidence for an inverse relation between birth weight and subsequent blood pressure (BP) later in life (3), most of which comes from animal research and human cohort studies (4). Low birth weight is associated with a reduction in nephron number in humans (5, 6), which may increase susceptibility to renal injury and thereby affect BP. Besides birth weight, elevated BP and adiposity in childhood are independent predictors of adult hypertension and cardiovascular disease (7).

In children, hypertension is defined as systolic or diastolic BP ≥95th percentile and elevated BP as values ≥90th percentile and <95th percentile (8). In a recent meta-analysis, the prevalence of hypertension and elevated BP among 2- to 19-y-old African children was 5.5% and 12.7%, respectively (9). Results from a meta-analysis and systematic review indicate that BP tracks from childhood into adulthood (10) and 1 study reported that even short periods of hypertension in childhood may increase the risk of hypertension as an adult (11). The prevalence of adult hypertension (defined as systolic BP ≥140 mm Hg or diastolic BP ≥90 mm Hg) in Ghana is relatively high, ranging between 19.3% and 54.6% among adults (12), which parallels the general trend towards chronic disease in a country undergoing the nutrition transition. The double burden of undernutrition in early life and subsequent exposure to risk factors for overweight puts young children at high risk of later development of chronic diseases. Interventions that can improve nutrition in early life may thus help to prevent these consequences.

The International Lipid-Based Nutrient Supplements (iLiNS)-DYAD trial in Ghana evaluated the effects of maternal and child supplementation with small-quantity lipid-based nutrient supplements (LNSs) on birth outcomes and child growth. As reported previously, LNS supplementation had a positive effect on birth weight (13) and weight and length by 18 mo of age (14). The current study reports results from a follow-up of the children from the iLiNS-DYAD trial at 4–6 y of age. The first objective of this analysis was to determine the effect of LNS supplementation on systolic BP (SBP) and diastolic BP (DBP). Because birth weight has been inversely related to BP in later life, we hypothesized that children in the LNS group would have lower BP than children in the control groups. The second objective was to examine the maternal and child factors related to BP in this cohort of preschool children, including maternal BMI and BP, and child anthropometric indexes, dietary variables, and physical activity.

## Methods

### Location and study design of the main trial

The iLiNS-DYAD-Ghana trial (NCT00970866) was conducted in the Yilo and Lower Manya Krobo districts of the Eastern Region of Ghana between December 2009 and March 2014. Details of the study are published elsewhere (13). Briefly, the study was a partially double-blind, randomized controlled trial that enrolled 1320 women 18 y or older at ≤20 weeks of gestation attending antenatal clinics in 4 main health facilities in the study area. The women were randomly assigned to 1 of 3 supplementation groups: *1*) daily iron and folic acid capsules during pregnancy, 200 mg/d calcium tablets (placebo) during the first 6 mo postpartum, and no infant supplementation; *2*) daily multiple micronutrient capsules (1–2 times the RDA of 18 vitamins and minerals) during pregnancy and the first 6 mo postpartum and no infant supplementation; or *3*) daily 20-g (118-kcal) LNSs during pregnancy and the first 6 mo postpartum followed by infant LNS supplementation from 6 to 18 mo of age. The maternal LNSs had the same micronutrient content as the multiple micronutrient supplement, plus calcium, magnesium, phosphorus, potassium, and macronutrients (essential fatty acids and a small amount of protein), whereas the infant LNSs had the same macronutrients and 22 micronutrients based on infant Recommended Nutrient Intakes (15). Women and children were followed ≤6 mo postpartum and 18 mo of age, respectively. Growth status of children was measured at birth, 6, 12, and 18 mo.

### Follow-up when children were 4–6 y of age

**FIGURE 1:**
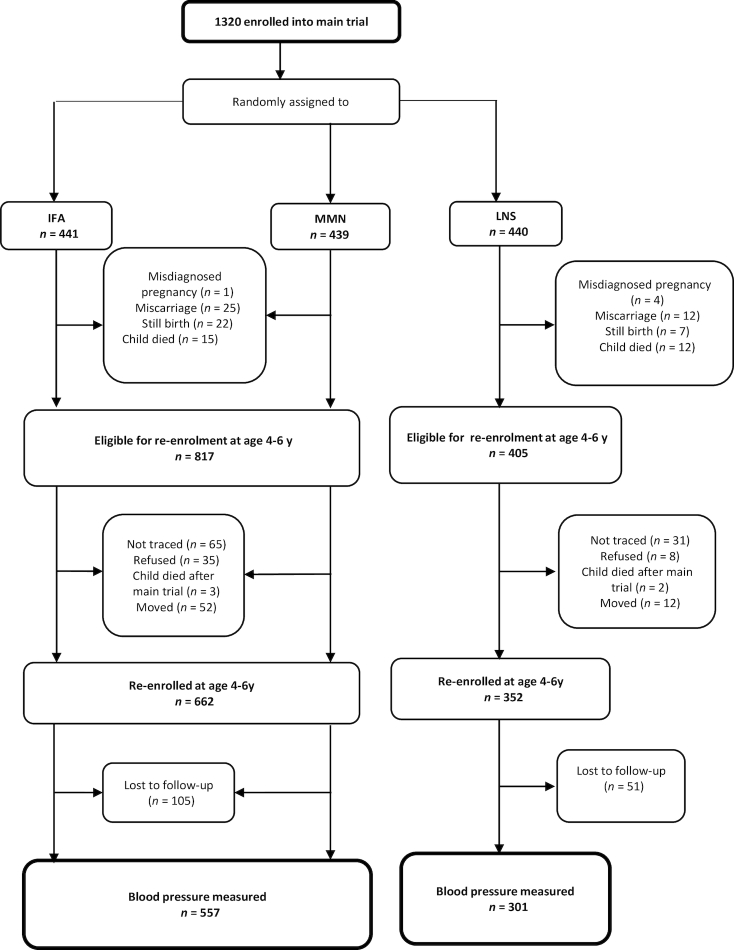
Study profile. IFA, iron and folic acid; LNS, lipid-based nutrient supplement; MMN, multiple micronutrients.

Participants in the follow-up study (also NCT00970866) were the children born to the pregnant women who were randomly assigned into the trial. In the main trial, when a mother delivered twins, one of the twins was randomly selected as the study child. All children who were alive at the time of the follow-up study were potentially eligible to participate. At follow-up, after excluding misdiagnosed pregnancies (*n* = 5), miscarriages and stillbirths (*n* = 66), and child deaths before the end of the main trial (*n* = 27), 1222 children were potentially eligible to participate. Details of the study profile are shown in **Figure 1**.

The follow-up study protocols were approved by the Institutional Review Board of University of California Davis; the Ethics Committee for the College of Basic and Applied Sciences at the University of Ghana; and the Ghana Health Service Ethical Review Committee. Written informed consent was given by children's primary caregivers before data collection.

#### Data collection procedures.

Participants were contacted using the last known address and contact number provided. Field staff called to set up an appointment and visited the caregiver to obtain informed consent for the follow-up activities. After consent had been given, participants were scheduled for the data collection visits, the last of which was the laboratory visit, which took place in a central location. BP measurements for mothers and children were done in duplicate by a trained nurse and her assistant using automated BP monitors (Riester ri-champion N, for children; Omron BP742N, for mothers) with age-appropriate arm cuffs. Measurements were done while the child was sitting with the child's arm at their chest level on a table and feet resting flat on a footstool. For mothers, measurements were done following the same procedure as for children except mothers had their feet on the floor. Participants rested (sitting) for ≥5 min before measurements were taken.

Anthropometric measurements were taken according to WHO standard procedures (16) by trained anthropometrists. Height was measured using a stadiometer (Seca 217) to the nearest 0.1 cm, weight using a scale (Seca 875) to the nearest 50 g. All measurements were taken in duplicate and in triplicate if the first 2 measurements differed by a predefined amount: 0.1 kg for weight and 0.5 cm for height.

Data on child food and beverage preferences and consumption, with an emphasis on sweet foods and beverages, were collected by administering a questionnaire to the caregiver (17). The questionnaire asked about the number of times different food and beverage items (or food and beverage groups) were consumed by the child in the week preceding the interview. The reported numbers of times for each of the items were summed up to obtain a score for total frequency of consumption of sweet foods and beverages.

Anthropometry, BP measurements, and recall of child food and beverage consumption were targeted for the full sample. Physical activity was to be estimated in a random subsample of 630 (*n* = 420 and 210, non-LNS and LNS, respectively) children. Of this number, 376 children were fitted with a device and physical activity was successfully estimated for 353 children (*n* = 224 and 129, non-LNS and LNS, respectively). Every child in the subsample was fitted with a single accelerometer (Actigraph GT3X) for 1 wk to estimate physical activity. Accelerometers were fitted to an elastic belt and fastened to the child's right hip by trained data collectors. Caregivers were instructed to let the child wear the accelerometer continuously day and night for the 1-wk period unless they experienced discomfort. Activity was stored in 60-s intervals or epochs. Physical activity data were analyzed using ActiLife data analysis software version 6.13.1 to generate mean vector magnitude accelerometer counts per minute per participant.

All data collection staff were blinded to group assignments. The data analyst remained blinded until all decisions regarding outliers for our first objective had been made.

#### Sample size and data analysis.

For these analyses, our sample size was the 858 children who had BP data at the 4–6 y follow-up. With this sample size we were powered to detect a difference of 1.07 mm Hg in systolic BP (an effect size of 0.11, based on the SD of 9.87) between the 2 groups (non-LNS and LNS) at 80% power.

For our first objective, to determine the effect of the intervention on BP (a secondary objective of the study), a statistical analysis plan was posted on the iLiNS Project website (www.ilins.org) before data analysis. The primary outcomes for this analysis were SBP and DBP and secondary outcomes were SBP and DBP *z* scores standardized based on gender, age, and height, as well as high BP defined as SBP or DBP ≥90th percentile of the reference population. BP percentiles and *z* scores were calculated using the equations given in the National Heart, Lung, and Blood Institute Fourth Report on the Diagnosis, Evaluation, and Treatment of High Blood Pressure in Children and Adolescents (8). Analysis was carried out based on the intention-to-treat principle using SAS version 9.4 (SAS Institute) and all tests were 2-sided and considered significant at the 5% level. Because our hypothesis was based on comparing the LNS group with the 2 non-LNS groups, the primary analysis for the first objective was a 2-group comparison. However, a sensitivity analysis was performed comparing all 3 groups. The effect of the intervention on continuous outcomes was examined using ANCOVA. Logistic regression was used for analysis of categorical outcomes. For all analyses, we controlled for child age at follow-up (minimally adjusted models). For fully adjusted models, several prespecified covariates were considered for inclusion in addition to child age: child sex, and maternal characteristics at enrollment in the main trial (gestational age, nulliparity, BMI, and household asset score). These additional covariates were included only if they were associated with an outcome at a 10% level of significance in bivariate analysis. Maternal SBP, DBP, and BMI at enrollment in the main trial, nulliparity, and child sex were tested as effect modifiers, and if the interaction term was significant (*P*-interaction <0.05) we conducted further analysis by stratifying the intervention groups on categories of the effect modifier. In addition, we conducted a per-protocol analysis by limiting the analysis to mothers who self-reported ≥80% adherence to the supplements during the pregnancy period.

**FIGURE 2:**
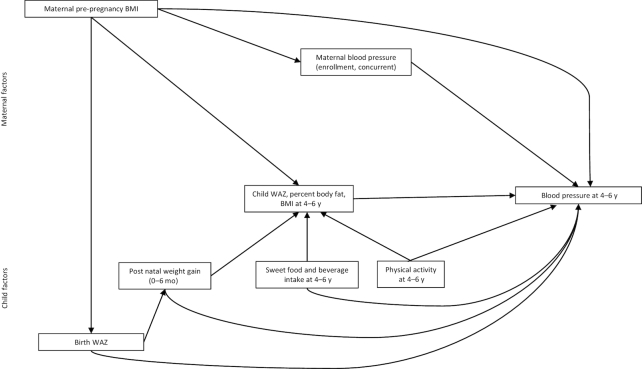
Conceptual model of maternal and child factors related to child blood pressure. WAZ, weight-for-age *z* score.

For the second objective, to identify the factors related to BP in this cohort of children, the analysis was based on the conceptual model in **Figure 2**. The outcomes were SBP, DBP, mean arterial pressure (MAP), and pulse pressure (PP) calculated as follows: MAP = DBP + [0.333 × (SBP − DBP)] and PP = SBP − DBP (18). We included MAP and PP because they may be helpful in interpreting the relative role of both arterial stiffness and vascular resistance in contributing to cardiovascular disease risk (19). We examined the relation of each of these outcomes to several maternal (maternal prepregnancy BMI, maternal BP at enrollment and follow-up) and child [birth WAZ (weight-for-age *z* score); postnatal weight gain (0–6 mo, i.e., difference between weight at 6 mo and birth weight); concurrent WAZ, percentage body fat, and BMI; sweet food and beverage intake at follow-up; physical activity at follow-up] factors. Estimated prepregnancy BMI was calculated from estimated prepregnancy weight (based on polynomial regression with gestational age, gestational age squared, and gestational age cubed as predictors) and height at enrollment (14). All variables were standardized before analysis of the data. For each model, the outcome and the factor were examined in an independent regression model adjusting for child age in minimally adjusted models and including in fully adjusted models other covariates (household asset score at enrollment, maternal age, education, and nulliparity at enrollment, child sex, and intervention group) if these covariates were associated with the outcome at a 10% level of significance in bivariate analysis. Household asset score was constructed based on ownership of a set of assets (radio, television, refrigerator, and stove), lighting source, drinking water supply, sanitation facilities, and flooring materials, developed into an index (with a mean of 0 and SD of 1) using principal components analysis (20). For each model, the value of the covariate for observations that had high leverage (>0.02) was truncated to the 2.5th or 97.5th percentile. We performed a sensitivity analysis with and without truncation of the observations and indicated where appropriate whether truncation made a difference to the results. Normality of the residuals was tested with the Shapiro–Wilk test and the linearity assumption was evaluated by plotting a residual against fit scatterplot. We also examined how the factors related to each other. First, for each path in the model, factor–outcome or factor–factor, we removed any path that was nonsignificant (*P* < 0.10). Second, for variables that were retained, we checked for mediation between independent variables and the outcome according to the model in Figure 2, using the binary mediation command in Stata version 15.0 (StataCorp) and we included any significant mediation pathways in the overall path model. The mediators tested were child WAZ and BMI *z* score at 4–6 y. Finally, for variables and pathways that were retained in the first 2 steps, the overall model coefficients were estimated using the SEM command in Stata specifying standardized coefficients and the maximum likelihood for missing values method to account for missing data. The overall path was modeled for only 1 outcome: child SBP.

## Results

Of the 1222 children eligible to participate in the follow-up study, we enrolled 662 and 352 in the non-LNS and LNS groups, respectively, and measured BP for 557 in the non-LNS and 301 in the LNS group (70% of live births from the main trial; 84.6% of re-enrolled children) by the end of the period for follow-up data collection (January to December, 2016). The reasons for nonparticipation were inability to trace the mother (*n* = 96), consent refusal (*n* = 43), child death after the main trial ended (*n* = 5), no longer residing in the study area (*n* = 64), and loss to follow-up after enrollment in the follow-up (*n* = 156). Details of the study flow are presented in Figure 1. There was a trend for a difference in proportion lost to follow-up between the groups (non-LNS: 36.7%; LNS: 31.6%, *P* = 0.07).

Maternal characteristics at enrollment into the main trial for women in the current analysis are shown in **Table 1**. More than 30% of the women were overweight (31% in the non-LNS group and 34% in the LNS group, *P* = 0.035) and >90% were married or cohabiting. The non-LNS group had a higher household assets score than the LNS group (0.07 compared with −0.11, *P* = 0.009) whereas the LNS women were heavier (61.5 compared with 62.9 kg, *P* = 0.035). About half the children were male and mean ± SD age was 5.0 ± 0.6 y. There were no significant differences in maternal enrollment characteristics between those included in this analysis (*n* = 858) and those lost to follow-up (**Supplemental Table 1**).

**TABLE 1:** Background characteristics of women and children in the iLiNS-DYAD-Ghana trial included in the follow-up^1^

	Non-LNS (*n* = 557)	LNS (*n* = 301)
Maternal characteristics at baseline		
Age, y	26.7 ± 5.4	26.7 ± 5.5
Gestational age at enrolment, wk	16.0 ± 3.2	16.1 ± 3.4
Formal education, y	7.6 ± 3.4	7.6 ± 3.7
Married or cohabiting, % (*n*/*N*)	93.5 (521/557)	92.7 (279/301)
Asset score^2^	0.07 ± 0.94	−0.11 ± 0.97
Nulliparous, % (*n*/*N*)	32.1 (179/557)	33.6 (101/301)
Weight, kg	61.5 ± 12.0	62.9 ± 12.4
Height, cm	158.8 ± 5.8	159.3 ± 5.4
Prepregnancy BMI,^3^ kg/m^2^	24.4 ± 4.5	24.8 ± 4.5
Overweight (BMI ≥ 25), % (*n*/*N*)	30.5 (166/545)	34.3 (102/297)
Systolic blood pressure, mm Hg	110.1 ± 10.8	110.7 ± 13.9
Diastolic blood pressure, mm Hg	62.3 ± 8.1	63.1 ± 9.3
Child characteristics		
Birth weight, g	2962.5 ± 425.9	3019.9 ± 415.9
Current age of child, y	5.0 ± 0.6	5.1 ± 0.6
Sex of child, % male	46.8	50.2
Weight at 4–6 y, kg	16.4 ± 2.1	16.7 ± 2.3
Height at 4–6 y, cm	106.1 ± 5.4	107.0 ± 5.9
Height-for-age *z* score at 4–6 y	−0.30 ± 0.91	−0.25 ± 0.99
BMI *z* score at 4–6 y	−0.57 ± 0.80	−0.55 ± 0.82
Fat mass at 4–6 y, %	15.3 ± 4.6	15.2 ± 5.0
Fat-free mass at 4–6 y, %	84.9 ± 5.5	84.8 ± 5.1

^1^Values are means ± SDs unless otherwise stated. iLiNS, international lipid-based nutrient supplements; LNS, lipid-based nutrient supplement group; non-LNS, IFA (iron and folic acid group) + MMN (multiple micronutrients group).

^2^Household asset score was constructed based on ownership of a set of assets and access to certain amenities using principal components analysis.

^3^Estimated prepregnancy BMI was calculated from estimated prepregnancy weight (based on polynomial regression with gestational age, gestational age squared, and gestational age cubed as predictors) and height at enrollment.

Mean ± SD SBP and DBP of children at 4–6 y were 99.0 ± 9.9 and 60.1 ± 6.7 mm Hg, respectively, and prevalence of high BP was 30.4%. Continuous and categorical BP outcomes adjusted for age are shown in **Tables 2** and **3**, respectively. There were no significant differences between the intervention groups for any of the BP outcomes. The aforementioned results did not change after adjusting for additional prespecified covariates. The sensitivity analysis revealed no significant differences in the 3-group comparisons for any of the continuous (**Supplemental Table 2**) or categorical (**Supplemental Table 3**) outcomes.

**TABLE 2:** Comparison of continuous BP measurements of children in the iLiNS-DYAD-Ghana trial follow-up at 4–6 y^1^

	Non-LNS (*n* = 557)	LNS (*n* = 300)	*P* value	Difference in mean (95% CI)
SBP, mm Hg	99.2 ± 0.4	98.5 ± 0.6	0.317	0.71 (−0.68, 2.09)
SBP *z* score	0.67 ± 0.04	0.59 ± 0.05	0.199	0.09 (−0.05, 0.22)
DBP, mm Hg	60.1 ± 0.3	60.0 ± 0.4	0.805	0.12 (−0.83, 1.07)
DBP *z* score	0.69 ± 0.03	0.69 ± 0.04	0.891	0.01 (−0.08, 0.09)

^1^Values represent means ± SEs and differences in means (95% CIs). Values in the table are adjusted for child age. Results are based on ANCOVA (SAS PROC GLIMMIX). BP, blood pressure; DBP, diastolic blood pressure; iLiNS, international lipid-based nutrient supplements; LNS, lipid-based nutrient supplements group; non-LNS, IFA (iron and folic acid group) + MMN (multiple micronutrients group); SBP, systolic blood pressure.

**TABLE 3:** Comparison of categorical BP outcomes of children in the iLiNS-DYAD-Ghana trial follow-up at 4–6 y^1^

	Non-LNS (*n* = 536)	LNS (*n* = 289)	*P* value	OR (95% CI)
Prehypertensive/hypertensive				
(SBP ≥ 90th percentile)				
Prevalence, %	26.3	22.2	0.146	0.78 (0.55, 1.09)
Prehypertensive/hypertensive				
(DBP ≥ 90th percentile)				
Prevalence, %	14.4	14.2	0.999	1.00 (0.66, 1.51)
Prehypertensive/hypertensive				
(SBP or DBP ≥ 90th percentile)				
Prevalence, %	31.7	28.0	0.251	0.83 (0.61, 1.14)

^1^Values are the percentage of participants whose response was “yes” for the outcome in question and OR (95% CI) obtained by comparing the groups. Reference = non-LNS group for all outcomes. Values in the table are adjusted for child age. Results are based on logistic regression (SAS PROC GLIMMIX). BP, blood pressure; DBP, diastolic blood pressure; iLiNS, international lipid-based nutrient supplements; LNS, lipid-based nutrient supplements group; non-LNS, IFA (iron and folic acid group) + MMN (multiple micronutrients group); SBP, systolic blood pressure.

Tests for interaction showed no significant (*P* < 0.05) interactions of intervention group with any of the following variables at baseline: maternal prepregnancy BMI, maternal SBP or DBP, nulliparity, and child sex (data not shown). When the analysis was restricted to mothers who reported ≥80% adherence during the pregnancy period (*n* = 316 in the non-LNS group and *n* = 134 in the LNS group), we observed a marginally significantly higher SBP in the non-LNS group than in the LNS group (99.6 ± 0.6  compared with 97.7 ± 0.9 mm Hg; *P* = 0.06), a borderline significant difference in SBP *z* score (0.70 ± 0.05 compared with 0.51 ± 0.08; *P* = 0.049), and a marginally significant difference in the prevalence of high BP (34.9% compared with 26.2%; *P* = 0.08). The results remained the same in the adjusted analysis (data not shown). There was no difference in any of the outcomes for the sensitivity analyses among high adherers (data not shown).

Summary statistics for the variables used in the analyses for objective 2 are presented in **Supplemental Table 4**. WAZ at 4–6 y was positively associated with sweet food and beverage intake [β (SE) = 0.07 (0.03), *P* = 0.018], WAZ at birth was inversely associated with postnatal weight gain [β (SE) = −0.07 (0.04), *P* = 0.07], and WAZ and BMI at 4–6 y were positively associated [β (SE) = 0.65 (0.02), *P* < 0.0001]. The relation between BMI at 4–6 y and sweet food and beverage intake was nonsignificant [β (SE) = 0.02 (0.03), *P* = 0.53].

**Table 4** shows the associations between each independent factor and each outcome, adjusting only for child age. SBP, DBP, PP, and MAP were all positively associated (*P* < 0.10) with postnatal weight gain, WAZ at 4–6 y, BMI *z* scores at 4–6 y, and maternal SBP and DBP at follow-up. In addition, child SBP and MAP were positively associated with WAZ at birth and sweet food and beverage intake, whereas child DBP and MAP were positively associated with maternal SBP and DBP at enrollment.

**TABLE 4:** Associations between outcomes (child SBP, DBP, PP, and MAP) and each factor in the iLiNS-DYAD-Ghana trial follow-up at 4–6 y^1^

	SBP		DBP		PP		MAP	
Factors	β (SE)	*P* value	β (SE)	*P* value	β (SE)	*P* value	β (SE)	*P* value
WAZ at birth	0.08 (0.04)	0.026	0.06 (0.04)	0.132	0.04 (0.04)	0.223	0.08 (0.04)	0.035
Postnatal weight gain (0–6 mo)^2^	0.10 (0.04)	0.004	0.06 (0.04)	0.085	0.08 (0.04)	0.035	0.09 (0.04)	0.012
WAZ at 4–6 y	0.29 (0.04)	<0.0001	0.23 (0.04)	<0.0001	0.16 (0.04)	<0.0001	0.29 (0.04)	<0.0001
BMI *z* score at 4–6 y	0.21 (0.04)	<0.0001	0.17 (0.04)	0.0002	0.12 (0.04)	0.006	0.20 (0.04)	<0.0001
Percentage fat mass at 4–6 y	−0.04 (0.04)	0.260	0.02 (0.04)	0.638	−0.05 (0.04)	0.145	−0.01 (0.04)	0.846
Physical activity^3^ at 4–6 y	0.04 (0.06)	0.465	0.05 (0.06)	0.345	0.01 (0.06)	0.802	0.06 (0.06)	0.331
Sweet food and drink intake	0.06 (0.03)	0.062	0.04 (0.03)	0.206	0.04 (0.03)	0.282	0.06 (0.03)	0.084
Maternal SBP at enrollment	0.05 (0.03)	0.124	0.08 (0.03)	0.016	−0.01 (0.03)	0.881	0.07 (0.03)	0.034
Maternal SBP at follow-up	0.13 (0.03)	0.0002	0.16 (0.03)	<0.0001	0.03 (0.03)	0.316	0.16 (0.03)	<0.0001
Maternal DBP at enrollment	0.05 (0.04)	0.130	0.13 (0.03)	0.0002	−0.04 (0.03)	0.233	0.10 (0.10)	0.002
Maternal DBP at follow-up	0.12 (0.03)	0.0008	0.16 (0.03)	<0.0001	0.02 (0.03)	0.593	0.15 (0.03)	<0.0001
Maternal prepregnancy^4^ BMI	0.04 (0.03)	0.260	0.05 (0.03)	0.184	0.01 (0.03)	0.882	0.05 (0.03)	0.185

^1^Values in the table are coefficients (SEs) from separate models of each independent factor with each outcome, adjusted only for child age. Coefficients are standardized. Results are based on regression (SAS PROC REG). DBP, diastolic blood pressure; iLiNS, international lipid-based nutrient supplements; MAP, mean arterial pressure; PP, pulse pressure, SBP, systolic blood pressure; WAZ, weight-for-age *z* score.

^2^Postnatal weight gain = weight at 6 mo − birth weight.

^3^Physical activity was estimated as vector magnitude count per minute.

^4^Estimated prepregnancy BMI was calculated from estimated prepregnancy weight (based on polynomial regression with gestational age, gestational age squared, and gestational age cubed as predictors) and height at enrollment.

**FIGURE 3:**
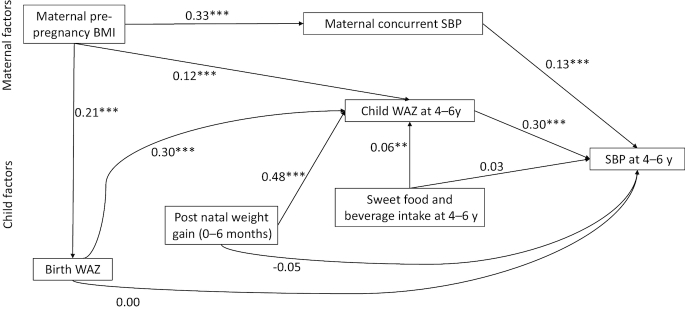
Final path model of the maternal and child factors related to child systolic blood pressure at 4–6 y. Numbers in the model are standardized β-coefficients. **P  > 0.0001 but *P* < 0.001; ***P < 0.0001. SBP, systolic blood pressure; WAZ, weight-for-age *z* score.

For the path analysis, we modeled the overall path only for SBP because of the 4 outcomes, the β-coefficients for the associations with the predictors were highest for SBP and MAP but SBP is measured more commonly than MAP. We also included maternal BP at follow-up and not enrollment because the enrollment measures were not significantly related to child SBP. Maternal SBP at enrollment and follow-up were, however, correlated with each other [correlation coefficient (*r*) = 0.47; *P* < 0.0001]. Child BMI *z* score and WAZ at 4–6 y were highly correlated (*r* > 0.65; *P* < 0.0001) but the β-coefficient for the association of child BMI *z* score with SBP was lower than that for WAZ, so BMI *z* score was dropped from the final model. In the final path model (**Figure 3**), WAZ at 4–6 y and maternal concurrent SBP remained positively associated with child SBP. Birth WAZ and postnatal weight gain were not significantly associated with child SBP in the path model even though both factors were significantly associated with child SBP in a regression analysis controlling only for child age. Maternal prepregnancy BMI was significantly associated with both child WAZ at follow-up (4–6 y) and maternal SBP at follow-up.

**FIGURE 4:**
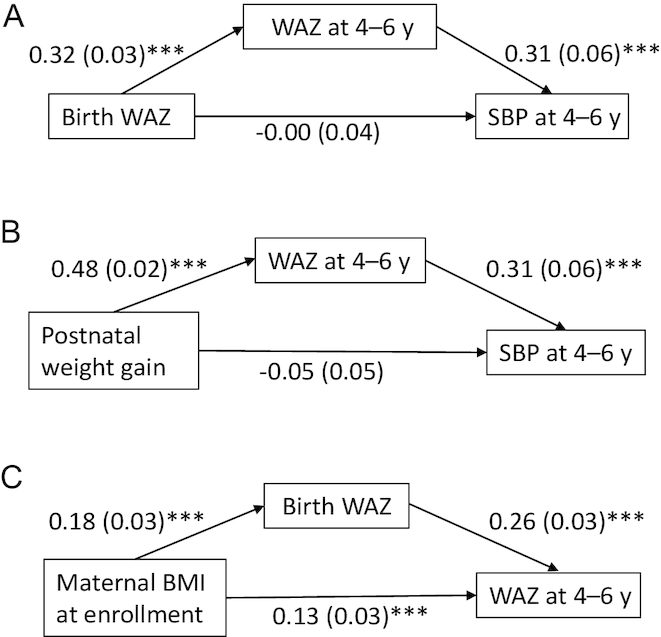
Path diagrams representing mediation analysis within the final path model. (A) Mediation between birth WAZ and SBP at 4–6 y by WAZ at 4–6 y. Parameter estimates of mediation: 0.08 (0.02)***; proportion mediated 97.2%. (B) Mediation between postnatal weight gain (0–6 mo) and SBP at 4–6 y by WAZ at 4–6 y. Parameter estimates of mediation: 0.13 (0.03)***; proportion mediated 75.6%. (C) Mediation between maternal prepregnancy BMI and WAZ at 4–6 y by birth WAZ. Parameter estimates of mediation: 0.07 (0.02)***; proportion mediated 25.9%. Numbers in the model were obtained from mediation analysis in STATA software version 15.0 and represent parameter estimates (SEs). ***P < 0.0001. SBP, systolic blood pressure; WAZ, weight-for-age *z* score.

Birth WAZ, postnatal weight gain, and sweet food and beverage intake were also positively associated with child WAZ at 4–6 y. WAZ at 4–6 y was a significant mediator of the relations between birth WAZ and child SBP and between postnatal weight gain and child SBP. **Figure 4**A illustrates the relation between birth WAZ and child SBP mediated by WAZ at 4–6 y. Birth WAZ was positively associated with WAZ at 4–6 y in a fully adjusted model. Child SBP was positively associated with WAZ at 4–6 y but not with birth WAZ. In Figure 4B we examine WAZ at 4–6 y as a mediator between postnatal weight gain and child SBP. Postnatal weight gain was positively associated with WAZ at 4–6 y whereas SBP at 4–6 y was inversely associated with postnatal weight gain but positively associated with WAZ at 4–6 y. Birth WAZ was a significant mediator of the relation between maternal prepregnancy BMI and WAZ at 4–6 y (Figure 4C). However, the direct association between maternal prepregnancy BMI and WAZ at 4–6 y also remained significant.

## Discussion

Because increased birth weight has been associated with lower BP later in life (21–30), and the provision of LNSs during pregnancy increased birth weight in the iLiNS-DYAD-Ghana trial (13), we hypothesized that children in the LNS group would have lower BP at 4–6 y than those in the non-LNS group. The primary results did not support this hypothesis: we observed no effect of LNSs provided to mothers prenatally and in the first 6 mo of lactation and to children from 6 to 18 mo of age on child BP in this follow-up of the iLiNS-DYAD-Ghana cohort. However, in the per-protocol analysis among high adherers (≥80% adherence during pregnancy), we observed lower SBP *z* score (*P* < 0.05), SBP, and prevalence of high BP (*P* < 0.10) in the LNS group. There was a high overall prevalence of high BP (30%) among the children. Child BP at 4–6 y was significantly associated with concurrently measured maternal SBP and child WAZ. Birth weight and postnatal weight gain (0–6 mo of age) were both related indirectly to child SBP via their positive associations with child WAZ at 4–6 y.

Strengths of our study include the large sample size and the blinding of field staff (study nurses and anthropometrists) to group assignment. A potential limitation is that loss to follow-up was somewhat higher in the non-LNS group. However, there were no differences in characteristics between the sample included in this analysis and those lost to follow-up, suggesting that our conclusions are generalizable to the full cohort. Another limitation is that the non-LNS group had a lower household asset score and a higher percentage of maternal overweight than the LNS group. However, we adjusted for asset score, maternal prepregnancy BMI, and other prespecified covariates in our analysis. A further limitation is that we did not measure certain dietary factors associated with BP, such as sodium intake. Although we did not measure BP by the recommended auscultation procedure (manual BP cuff and stethoscope), we used a validated oscillometric device (automated BP cuff). We also used appropriately sized cuffs (child and adult) for the measurement of BP and allowed participants to rest for ≥5 min before measurements were taken to prevent artificially elevated values.

Our primary results are consistent with those of a previous trial providing both prenatal and postnatal nutrition supplementation that included a long-term follow-up: the INCAP Longitudinal Study in Guatemala (31). The investigators found no independent effect of the supplements (Atole compared with Fresco, intervention compared with control supplement) on SBP or DBP in young adulthood. Results of other follow-up studies, in which the intervention included only prenatal supplementation (with food or micronutrients) (32–38), have been mixed. Two trials in Nepal examined the effect of maternal micronutrient supplementation on offspring BP (33, 37, 38). One showed lower SBP but not DBP in the multiple micronutrient group than in the control group when the children were 2.5 y of age (38), but no difference at 8.5 y (33). The other trial showed no difference in BP when children were ∼7.5 y of age (37). Four other trials involved prenatal food supplementation (with or without micronutrient supplements) (34, 36) or prenatal calcium supplementation (32, 35); 3 indicated no effect on child BP, but 1 of the calcium trials (32) showed lower SBP and lower risk of hypertension in the calcium group than in the placebo group. Although we cannot directly compare the results of these trials to ours because of differences in study design, all except the latter trial agree with our results showing no main effect of prenatal or postnatal supplementation on later BP. It is important to note that the increase in birth weight observed in the LNS group in the main iLiNS-DYAD-Ghana trial was <100 g, whereas the magnitude of the association between birth weight and later BP reported in observational studies was a decrease of 1–4 mm Hg in BP for every 1-kg increase in birth weight (25, 26, 30). Thus, the increase in birth weight resulting from prenatal nutrition interventions may not be large enough to lead to a detectable difference in BP later in life.

The prevalence of high BP was 30% in this cohort, which is similar to the prevalence observed in a Spanish population of 4- to 6-y-old children: 27.5% in boys and 30.6% in girls (18). In a study of 5- to 8-y-old children in Brazil, the investigators used 2 different definitions for prevalence of prehypertension/hypertension, based on elevated SBP (35%) or elevated DBP (5%) (39). When we considered prehypertension/hypertension due only to elevated SBP, prevalence was 25% (data not shown) in our cohort, which is lower than in Brazil (39), but when based only on elevated DBP, prevalence was 14%, higher than the 5% rate reported in Brazil.

We did not observe an inverse relation between birth weight and SBP in this cohort as has been reported in several prospective cohort studies (21, 22, 25, 26, 28–30) and a systematic review (3). Rather, we found a positive relation between birth WAZ and BP at 4–6 y in regression analysis controlling only for child age. Similar results have been reported for an Aboriginal community where low birth weight individuals had lower weight, height, BMI, cholesterol concentration, and BP at 20–38 y compared to normal birth weight individuals (40) and in the Pelotas, Brazil birth cohort where SBP and DBP at 11 y were positively associated with birth weight and length (41). However, in the Pelotas cohort, further adjustment for several covariates and current BMI resulted in an inverse association of birth weight with SBP. When we adjusted for WAZ or BMI at 4–6 y the relation between birth WAZ and child SBP did not become inverse. Thus, our results do not support the hypothesis that low birth weight is a risk factor for hypertension later in life. The mean birth weight in our study was 3.0 kg, comparable to the mean birth weight of ≥3 kg in most of the studies (22, 26, 28–30) reporting an inverse association between birth weight and BP, except for 2 studies with lower mean birth weights (21, 25). However, in our cohort the prevalence of low birth weight was low and at 4–6 y the prevalence of overweight was low, which may explain why we did not observe an inverse association between birth weight and BP. In addition, although the prevalence of overweight in our cohort was low, the prevalence of elevated BP was high, suggesting that in this population factors other than body size may also be critical determinants of BP.

We observed a positive association between postnatal weight gain (0–6 mo) and all BP outcomes at 4–6 y in regression analysis controlling only for age, similar to the findings of the Manchester Children's Growth and Vascular Health study (42), which showed that change in weight from birth to 3 mo of age in children of South Asian and Caucasian origin was positively associated with SBP at 12 mo of age. A similar result was also observed in a prospective cohort in Amsterdam, in which faster relative weight gain after the first month was associated with higher SBP and DBP at 5 y of age (43).

We found that child WAZ at 4–6 y of age largely mediated the associations of both birth WAZ and postnatal weight gain with BP at 4–6 y, suggesting that current size is a more important determinant than body size or growth in infancy. In addition to WAZ, BMI *z* score at 4–6 y was positively associated with BP in a regression analysis controlling only for age, but the association with BMI *z* score was not significant when WAZ was taken into account. Other studies have also reported a positive association between BMI and BP in children (28, 44, 45). In children and adolescents, the normal range of BP depends on body size and age (8). We did not observe a relation between percentage body fat and BP in this cohort of children, although BMI was associated with BP, suggesting that the association is driven by overall weight. In the Amsterdam Born Child and their Development study, which examined ethnic differences in the relations of children's BP to BMI, waist-to-height ratio, and fat mass index, the strongest association was observed for BMI, and the associations of BP with BMI and fat mass index differed by ethnicity (46).

We also found a positive association between maternal BP (at both baseline and follow-up) and child BP, although the association was stronger with maternal BP at follow-up. In the Generation R prospective cohort study in the Netherlands (47), maternal BP at all time points in pregnancy (early, mid-, and late pregnancy) was positively associated with childhood BP at 6 y, with the strongest association observed with early pregnancy maternal SBP. A similar result was observed in the Avon Longitudinal Study of Parents and Children in the United Kingdom, in which higher maternal BP in early pregnancy was associated with higher child BP at 7 y (48). The study investigators concluded that the observed association may be driven by shared genetic or environmental factors.

In the final path model accounting for all factors, there was no direct relation between SBP and either birth WAZ or postnatal weight gain. These 2 early-life factors operated through current WAZ, which was the strongest factor directly related to BP at preschool age. Postnatal weight gain was a stronger predictor of current WAZ than was birth WAZ. Sweet food and drink consumption at 4–6 y and maternal prepregnancy BMI were also positively related to current WAZ, and maternal prepregnancy BMI was also related to maternal BP. We did not observe a direct relation between maternal prepregnancy BMI and child SBP but we observed a significant relation between WAZ at age 4–6 y and child SBP. A recent systematic review (49) suggested that the relation between maternal prepregnancy body weight and offspring BP may be an indirect effect mediated through offspring anthropometric status, as we observed. To further understand the relation between maternal prepregnancy BMI and WAZ at age 4–6 y, we tested birth WAZ as a mediator of the relation. Birth WAZ was a significant mediator but the direct relation between maternal prepregnancy BMI and WAZ at 4–6 y also remained significant, indicating the relation between maternal prepregnancy BMI and WAZ at 4–6 y is not solely driven by WAZ at birth.

In conclusion, we did not find a difference in BP between the intervention groups at the 4–6 y follow-up in the iLiNS-DYAD-Ghana cohort, which is consistent with findings from previous trials. Our results indicate that this preschool population is at high risk of hypertension in later life. Child weight and maternal BP were directly and significantly related to child BP. Given that BP tends to remain consistent from childhood to adulthood, further follow-up of this population would be informative. These results suggest the need to evaluate the environmental and lifestyle factors that increase the risk of noncommunicable diseases in this population, in order to design effective intervention strategies.

## Supplementary Material

nxy285_Supplemental_Files

## Notes

Supported by a Bill & Melinda Gates Foundation grant (# OPP49817) to the University of California, Davis.

Author disclosures: SMK, SA-A, RRY, BMO, SMT, MEO, HO, ELP, and KGD, no conflicts of interest.

Supplemental Tables 1–4 are available from the “Supplementary data” link in the online posting of the article and from the same link in the online table of contents at https://academic.oup.com/jn/.

Abbreviations used: BP, blood pressure; DBP, diastolic blood pressure; iLiNS, International Lipid-Based Nutrient Supplements; LNS, lipid-based nutrient supplement; MAP, mean arterial pressure; PP, pulse pressure; SBP, systolic blood pressure; WAZ, weight-for-age *z* score.
