# Supplementary material for: Maternal–Infant Supplementation with Small-Quantity Lipid-Based Nutrient Supplements Does Not Affect Child Blood Pressure at 4–6 Y in Ghana: Follow-up of a Randomized Trial
Source: J Nutr. 2019 Feb 11;149(3):522–31. doi: 10.1093/jn/nxy285 (PMC6398380; doi:10.1093/jn/nxy285)
Supplement: nxy285_Supplemental_Files [file nxy285_supplemental_files.zip › Online Supporting Material Oct 10 Table 1.pdf]

Supplemental Table 1: Background characteristics of women included in the International Lipid-Based Nutrient Supplements (iLiNS)-DYAD Ghana trial follow-up analysis at 4-6 y and those not included[1]

**Variable**	**In the follow-up [*n*=858]**	**Not in the follow-up [*n*=462]**	***P***
**Maternal Characteristics**			
Age (y)	26.7 ± 5.4	26.7 ± 5.7	0.929
Gestational age at enrolment (wk)	16.1 ± 3.3	16.2 ± 3.2	0.324
Years of formal education	7.6 ± 3.5	7.7 ± 3.9	0.749
Married or Cohabiting (% [*n*])	93.2 [800/858]	91.8 [423/461]	0.323
Asset score	0.01 ± 0.95	0.01 ± 1.07	0.894
Nulliparous women (% [*n*])	32.6 [280/858]	36.4 [166/456]	0.170
Weight (kg)	62.0 ± 12.2	61.8 ± 11.5	0.770
Height (cm)	159.0 ± 5.7	158.5 ± 5.7	0.131
Pre-pregnancy BMI (kg/m^2)	24.5 ± 5.0	24.5 ± 4.1	0.891
Overweight (BMI ≥ 25) (% [*n*])	31.8 [268/842]	31.2 [140/449]	0.355
Systolic blood pressure (mmHg)	110.4 ± 12.0	110.6 ± 11.7	0.706
Diastolic blood pressure (mmHg)	62.6 ± 8.5	62.7 ± 8.2	0.716
**Child Characteristics**			
Sex of child, (% male)	48.0	49.0	0.847
Birth weight (kg)	3.0 ± 0.4	3.0 ± 0.5	0.928

[1]Values are mean ± SD unless otherwise stated

Estimated pre-pregnancy BMI was calculated from estimated pre-pregnancy weight (based on polynomial regression with gestational age, gestational age squared, and gestational age cubed as predictors) and height at enrollment
