# Supplementary material for: Maternal–Infant Supplementation with Small-Quantity Lipid-Based Nutrient Supplements Does Not Affect Child Blood Pressure at 4–6 Y in Ghana: Follow-up of a Randomized Trial
Source: J Nutr. 2019 Feb 11;149(3):522–31. doi: 10.1093/jn/nxy285 (PMC6398380; doi:10.1093/jn/nxy285)
Supplement: nxy285_Supplemental_Files [file nxy285_supplemental_files.zip › Online Supporting Material Oct 10 Table 2.pdf]

Supplemental Table 2: Blood pressure measurements of children in the International Lipid-Based Nutrient Supplements (iLiNS)-DYAD Ghana trial follow-up at 4-6 y by 3 groups (IFA, MMN, LNS)[1]

	**IFA [*n*=261]**	**MMN [*n*=296]**	**LNS [*n*=301]**	***P***
Systolic blood pressure (mmHg)	99.5 ± 0.6	98.9 ± 0.6	98.5 ± 0.6	0.460
SBP z-score	0.73 ± 0.06	0.62 ± 0.05	0.59 ± 0.05	0.183
Diastolic blood pressure (mmHg)	60.0 ± 0.4	60.3 ± 0.4	60.0 ± 0.4	0.843
DBP z-score	0.69 ± 0.04	0.69 ± 0.04	0.69 ± 0.04	0.988

[1]Values represent mean ± SE. Results are based on ANCOVA (SAS PROC GLIMMIX). IFA, Iron + Folic Acid tablet; MMN, Multiple Micronutrient tablet; LNS, Lipid-based Nutrient Supplements; SBP, Systolic blood pressure; DBP, Diastolic blood pressure
