# Supplementary material for: Maternal–Infant Supplementation with Small-Quantity Lipid-Based Nutrient Supplements Does Not Affect Child Blood Pressure at 4–6 Y in Ghana: Follow-up of a Randomized Trial
Source: J Nutr. 2019 Feb 11;149(3):522–31. doi: 10.1093/jn/nxy285 (PMC6398380; doi:10.1093/jn/nxy285)
Supplement: nxy285_Supplemental_Files [file nxy285_supplemental_files.zip › Online Supporting Material Oct 10 Table 3.pdf]

Supplemental Table 3: Prevalence of risk of high blood pressure in the International Lipid-Based Nutrient Supplements (iLiNS)-DYAD Ghana trial at 4-6 y by 3 groups (IFA, MMN, LNS)[1]

	**IFA [*n*=255]**	**MMN [*n*=281]**	**LNS [*n*=289]**	***P***
Pre-hypertensive/Hypertensive (SBP ≥90th percentile) Prevalence (%)	28.2	24.6	22.2	
OR (95% CI)		0.81 (0.55, 1.91)	0.70 (0.47, 1.03)	0.190
Pre-hypertensive/Hypertensive (DBP ≥90th percentile) Prevalence (%)	14.1	14.6	14.2	
OR (95% CI)		1.05 (0.65, 1.71)	1.03 (0.63, 1.67)	0.978
Pre-hypertensive/Hypertensive (SBP or DBP ≥90th percentile) Prevalence (%)	32.6	31.0	28.0	
OR (95% CI)		0.92 (0.64, 1.33)	0.80 (0.55, 1.15)	0.470

[1]Values are the percentage of participants whose response was “yes” for the outcome in question and OR (95% CI) obtained by comparing the groups. Reference = IFA group for all outcomes. Values in the table are adjusted for child age. Results are based on logistic regression (SAS PROC GLIMMIX). IFA, Iron + Folic Acid tablet; MMN, Multiple Micronutrient tablet; LNS, Lipid-based Nutrient Supplements; SBP, Systolic blood pressure; DBP, Diastolic blood pressure
