# Supplementary material for: Maternal–Infant Supplementation with Small-Quantity Lipid-Based Nutrient Supplements Does Not Affect Child Blood Pressure at 4–6 Y in Ghana: Follow-up of a Randomized Trial
Source: J Nutr. 2019 Feb 11;149(3):522–31. doi: 10.1093/jn/nxy285 (PMC6398380; doi:10.1093/jn/nxy285)
Supplement: nxy285_Supplemental_Files [file nxy285_supplemental_files.zip › Online Supporting Material Oct 10 Table 4.pdf]

Supplemental Table 4: Summary statistics for outcomes and child and maternal factors[1]

Variables	*n*	
**Outcomes at 4-6 y**		
Systolic blood pressure (mmHg)	858	99.0 ± 9.9
Diastolic blood pressure (mmHg)	858	60.1 ± 6.7
Pulse pressure (mmHg)	858	38.9 ± 7.9
Mean arterial pressure (mmHg)	858	73.0 ± 7.0
**Child factors**		
Weight-for-age z-score (WAZ) at birth	826	-0.66 ± 0.96
Postnatal weight gain (0-6 months) [kg]	758	4.3 ± 0.9
WAZ at 4-6 y	852	-0.71 ± 0.85
BMI z-score at 4-6 y	852	-0.56 ± 0.81
Percent fat mass at 4-6 y (%)	824	15.3 ± 4.7
Physical activity at 4-6 y (vector magnitude count)	315	1384 ± 246
Sweet food and drink intake at 4-6 y (number of times/wk)	848	16.3 ± 10.4
Low birth weight [*n* (%)]	826	93 (11.3)
**Maternal factors**		
Pre-pregnancy BMI (kg/m^2)	842	24.5 ± 4.5
Maternal systolic blood pressure at enrollment (mmHg)	857	110.4 ± 12.0
Maternal systolic blood pressure at follow-up (mmHg)	816	114.7 ± 15.5
Maternal diastolic blood pressure at enrollment (mmHg)	857	62.6 ± 8.5
Maternal diastolic blood pressure at follow-up (mmHg)	816	73.8 ± 11.3

[1]Values are mean ± SD or frequency (percentage) unless otherwise stated

Estimated pre-pregnancy BMI was calculated from estimated pre-pregnancy weight (based on polynomial regression with gestational age, gestational age squared, and gestational age cubed as predictors) and height at enrollment
